# Glycoproteins From *Rabdosia japonica* var. *glaucocalyx* Regulate Macrophage Polarization and Alleviate Lipopolysaccharide-Induced Acute Lung Injury in Mice *via* TLR4/NF-κB Pathway

**DOI:** 10.3389/fphar.2021.693298

**Published:** 2021-07-21

**Authors:** An-qi Ren, Hui-jun Wang, Hai-yan Zhu, Guan Ye, Kun Li, Dao-feng Chen, Tao Zeng, Hong Li

**Affiliations:** ^1^ Department of Pharmacology, School of Pharmacy, Fudan University, Shanghai, China; ^2^ The MOE Key Laboratory for Standardization of Chinese Medicines and the SATCM Key Laboratory for New Resources and Quality Evaluation of Chinese Medicines, Institute of Chinese Materia Medica, Shanghai University of Traditional Chinese Medicine, Shanghai, China; ^3^ Department of Biological Medicines and Shanghai Engineering Research Center of Immuno Therapeutics, School of Pharmacy, Fudan University, Shanghai, China; ^4^ Central Research Institute, Shanghai Pharmaceuticals Holding Co., Ltd., Shanghai, China; ^5^ Department of Pharmacognosy, School of Pharmacy, Fudan University, Shanghai, China; ^6^ Clinical Trial Institution, Obstetrics and Gynecology Hospital of Fudan University, Shanghai, China

**Keywords:** *Rabdosia japonica* var *glaucocalyx*, glycoproteins, acute lung injury, macrophage polarization, inflammation, TLR4/NF-κB signal pathway

## Abstract

**Background and Aims:** *Rabdosia japonica* var. *glaucocalyx* is a traditional Chinese medicine (TCM) for various inflammatory diseases. This present work aimed to investigate the protective effects of *R. japonica* var. *glaucocalyx* glycoproteins on lipopolysaccharide (LPS)-induced acute lung injury (ALI) and the potential mechanism.

**Methods:** Glycoproteins (XPS) were isolated from *R. japonica* var. *glaucocalyx*, and homogeneous glycoprotein (XPS5-1) was purified from XPS. ANA-1 cells were used to observe the effect of glycoproteins on the secretion of inflammatory mediators by enzyme-linked immunosorbent assay (ELISA). Flow cytometry assay, immunofluorescence assay, and Western blot analysis were performed to detect macrophage polarization *in vitro*. The ALI model was induced by LPS via intratracheal instillation, and XPS (20, 40, and 80 mg/kg) was administered intragastrically 2 h later. The mechanisms of XPS against ALI were investigated by Western blot, ELISA, and immunohistochemistry.

**Results:** *In vitro*, XPS and XPS5-1 downregulated LPS-induced proinflammatory mediators production including tumor necrosis factor-α (TNF-α), interleukin-1β (IL-1β), IL-6, and nitric oxide (NO) and upregulated LPS-induced IL-10 secretion. The LPS-stimulated macrophage polarization was also modulated from M1 to M2. *In vivo*, XPS maintained pulmonary histology with significantly reducing protein concentration and numbers of mononuclear cells in bronchoalveolar lavage fluid (BALF). The level of IL-10 in BALF was upregulated by XPS treatment. The level of cytokines including TNF-α, IL-1β, and IL-6 was downregulated. XPS also decreased infiltration of macrophages and polymorphonuclear leukocytes (PMNs) in lung. XPS suppressed the expression of key proteins in the TLR4/NF-κB signal pathway.

**Conclusion:** XPS was demonstrated to be a potential agent for treating ALI. Our findings might provide evidence supporting the traditional application of *R. japonica* var. *glaucocalyx* in inflammation-linked diseases.

## Introduction

*Rabdosia* genus is an important genus of the Labiatae family, with about 150 species worldwide (Huang et al., 2018). *Rabdosia japonica* (Burm. f.) Hara var. *glaucocalyx* (Maxim.) is a synonym of *Isodon japonicus* var. *glaucocalyx* (Maxim.) H.W.Li. *R. japonica* var. *glaucocalyx*, distributed mainly in northern China, is a traditional Chinese medicine (TCM) for hepatitis, gastritis, mastitis, and cough. It is recorded in Chinese Materia Medica (Hanson, 2011).

Previous studies focused a lot on organic extracts diterpenoids or phenolic compounds isolated from this plant. These active ingredients showed antioxidant, antitumor, and anti-inflammatory activities in treating neuroinflammation and gastritis (Hanson, 2011). Glycoproteins, as a kind of water-soluble compounds formed by carbohydrates and protein, possess the activities of modulating immunity (Fu et al., 2021) (Niu et al., 2020). The glycoproteins of *R. japonica* var. *glaucocalyx* (XPS) were isolated from the aqueous extracts of this plant, and two homogeneous glycoproteins (XPS5-1 and XPS10-1) were purified from XPS (Wang et al., 2020a). Our previous research found that XPS5-1 and XPS10-1 had antitumor activity *in vitro* (Wang et al., 2020a) but their anti-inflammatory effect remained unclear. According to our preliminary experiment, only XPS and XPS5-1 showed promising anti-inflammatory activity *in vitro*, and its anti-inflammatory mechanisms have not been reported yet*.*

Acute lung injury (ALI) is a kind of respiratory disorder with high mortality and calls for researches on effective drugs. The lung injury is characterized by excessive accumulation of inflammatory cells, uncontrolled secretion of cytokines, and damages of the alveolar-capillary membrane including epithelium and endothelium (Ge et al., 2020). Tentative clinical treatments including low tidal volume ventilation, modulation of immune defense, or fluid-conservative therapy are inefficient to ALI (Nieman et al., 2020). It was reported that lipopolysaccharide (LPS) could cause ALI (Ye and Liu, 2020) through the induction of innate immune response and inflammation (Huang et al., 2019). An animal model of ALI was established by intratracheal instillation of LPS in mice.

LPS activates TLR4, a member of the toll-like receptors (TLRs) family (Zhou et al., 2019). After recognizing LPS, the TLR4-linked nuclear transcription factor-κB (NF-κB) pathway can be activated through MyD88- and TRIF-dependent pathways (Wang et al., 2020c). The activated MyD88 pathway will upregulate the inflammatory cytokines (Han et al., 2018). High-level cytokines secreted by monocyte-macrophages in lungs severely affect pulmonary gas exchange and aggravate ALI by initiating, amplifying, and prolonging the inflammatory response (Liu J. et al., 2019). Reducing proinflammatory cytokines and increasing anti-inflammatory cytokines are at the heart of processes mediating lung inflammation (Watanabe et al., 2019).

The lung injury induced by LPS is associated with the lung excessive inflammation, which causes the activation of alveolar macrophage polarization homeostasis (Lin et al., 2020). Alveolar macrophages, as a kind of high heterogeneity and plasticity innate immunocyte, play an important role in the response to pneumonia and tissue repair (Zhou et al., 2020). Macrophages are generally grouped into classically activated (M1) macrophages and alternatively activated (M2) macrophages (Orecchioni et al., 2019). An increased M1 macrophage ratio, recruitment of neutrophils, and secretion of a large number of cytokines were observed in the acute inflammatory phase of ALI (Lin et al., 2020). M1 macrophages are found to exacerbate pathological inflammation, while M2 macrophages reduce autophagy and accelerate tissue repair (Su et al.). The dynamic changes of subsets balance and function of M1/M2 macrophages have a significant effect on inflammatory response (Zhou et al., 2020). Studies have indicated that compounds, which regulate the macrophages’ polarization, may reduce the lung injury through anti-inflammatory effects (Lin et al., 2020).

Here, the anti-inflammatory activity of XPS and XPS5-1 was first elucidated in ANA-1 cells (a murine macrophage cell line)*.* Then, the beneficial effect of XPS on ALI mice was explored to seek the effective drug for ALI.

## Materials and Methods

### Materials and Reagents

The commercial herb of *R. japonica* var. *glaucocalyx* was obtained from Huqingyutang Pharmaceutical Co., Ltd. (Hangzhou, China) by Prof. L.H.Wu from the Institute of Chinese Materia Medica, Shanghai University of Traditional Chinese Medicine, serving to authenticate. Monosaccharide standards (l-rhamnose, d-arabinose, d-xylose, d-mannose, d-glucose, d-galactose, and d-galacturonic acid) were obtained from Sigma-Aldrich (St. Louis, MO, United States of America). Diethylaminoethyl (DEAE) cellulose column and Superdex-200 were purchased from GE Healthcare Bio-Sciences AB (Uppsala, Sweden).

LPS (*Escherichia coli* O127:B8), thiazolyl blue tetrazolium bromide (MTT), and dexamethasone (DEX) were purchased from Sigma, United States, for the *in vitro* experiments. RPMI 1640 and FBS were bought from Gibco, America. Dimethyl sulfoxide (DMSO) was purchased from Merck Millipore, Germany. Fluorescently labeled antibodies against mouse CD11b, F4/80, CD86, and CD206 were obtained from Biolegend (San Diego, CA, United States). Arginase-1 (Cat. No. 93668), inducible nitric oxide synthase (iNOS) (Cat. No. 13120), and secondary antibodies (Cat. No. 4412 and 8889) were bought from CST Company (Beverly, MA, United States). DAPI was obtained from Dalian Meilun Biotechnology Co., Ltd. (China).

LPS (*Escherichia coli* O55:B5) was purchased from Sigma, United States, for the *in vivo* experiments. The ELISA kits were purchased from Boatman (China). Dexamethasone sodium phosphate injection (DEX) was purchased from Jiangxi Province Chuang Xin Pharmaceutical Group Co., Ltd. for the *in vivo* experiments. The ELISA kit for myeloperoxidase (MPO) was purchased from Lianke, Shanghai. Anti-mouse Ly-6G/Ly-6C (Cat. No. 108402) was purchased from Biolegend, United States. F4/80 (Cat. No. ab111101) was purchased from Abcam, Britain. Western blot kits were obtained from Beyotime (Shanghai, China). Antibody TLR4 (Cat. No. ab13556), TRAF6 (Cat. No. ab40675), and MyD88 (Cat. No. ab28763) were obtained from Abcam, Britain. GAPDH (Cat. No. AP0063) was purchased from Bioworld, China. Polyclonal antibodies NF-κB p65 (Cat. No. D14E12) and NF-κB p-p65 (Cat. No. S536) were bought from CST Company (Beverly, MA, United States).

### Plant Material and Extract Preparation

#### Isolation and Purification of XPS

The overground part of *R. japonica* var. *glaucocalyx* (5.0 kg) was extracted with 50 L water at 100°C for 3 h and three times. The supernatant of water extract was combined and concentrated under reduced pressure. After centrifugation, four times 95% ethanol was added and the precipitates were collected and freeze-dried to obtain XPS (315 g, yield rate 6.3%) (Wang et al., 2020 b; Wang et al., 2020c). XPS (5 g) was dissolved in 40 ml water and centrifuged. The residue was collected and centrifuged. The supernatant was combined and fractionated on a DEAE-cellulose column (50 cm × 5 cm). The XPS was eluted stepwise with distilled water, 0.1, 0.2, 0.5, and 1.0 M NaCl, to give XPSW, XPS1, XPS2, XPS5, and XPS10. XPS5 was further separated on a column of superdex-75 and eluted with 0.2 M NaCl to give XPS5-1 (41 mg, yield rate 0.82% from XPS) (Wang et al., 2020a). The total carbohydrate and protein contents were determined by the phenol-sulfuric acid and bicinchoninic acid (BCA) methods, respectively.

#### Homogeneity and Molecular Weight

High-performance gel permeation chromatography (HPGPC) was performed with Agilent 1,260 instrument fitted with the GPC software and the determination of homogeneity and molecular weights of the glycoproteins sample. The system was linked to the series-connected serial column of Ultrahydrogel 1,000 and Ultrahydrogel 250, ID 7.8 mm, and length 300 mm (Waters, United States), and the sample was eluted with 0.2 M NaCl at a flow rate of 0.80 ml/min at 40.0 ± 0.1°C. The gel filtration column was calibrated by pullulans with known molecular weight. The column temperature was kept at 40.0 ± 0.1°C. NaCl (0.2 M) was used as an eluant and the flow rate was kept at 0.8 ml/min. The sample concentration upon injection was 2 mg/ml and approximately 10 μL was injected (Wang et al., 2015).

#### Monosaccharide Composition Analysis

The sample was hydrolyzed with 2.0 M trifluoroacetic acid (TFA) at 121°C for 2 h. TFA was completely removed by repeating evaporation with methanol. Water was added to dissolve the residue and reduced by NaBH_4_ for 3 h at room temperature. After neutralization with acetic acid and evaporation to dryness, the residue was acetylated with acetic anhydride for 1 h at 100°C. The resulting alditol acetates were analyzed by GC-MS (Chaidedgumjorn et al., 2002).

### *In Vitro* Anti-Inflammatory Activity of XPS and XPS5-1 in LPS-Stimulated ANA-1 Macrophage

#### Cell Culture and *In Vitro* Treatment

ANA-1 cells (ATCC, America) are a murine macrophage cell line for *in vitro* experiments. Cells were cultured in 10% FBS-RPMI 1640 medium (Geng et al., 2020). The cells were plated as 1×10^6^ cells/mL, were cultured as 100 μL/well in a 96-well plate for 24 h, and then were treated with compounds with or without LPS for another 24 h (*n* = 3 for each group).

#### Cell Viability and Cytokines Assay

The cells were treated with XPS (25, 50, and 100 μg/ml) or XPS5-1 (100 μg/ml) for 24 h and were stimulated by LPS (0.5 μg/ml) simultaneously. DEX (20 μM) was administrated as a positive control. All the supernatant was collected for nitric oxide (NO) and other inflammatory cytokines’ testing. The cell viability was tested by MTT and was presented as a percentage of viable cells to the control group (Su et al.).

The production of NO was represented by its end product, nitrite in the cell culture supernatant. The supernatant was mixed with Griess reagent and shook for 15 min. The absorbance was recorded at 540 nm. The levels of TNF-α, IL-10, IL-1β, and IL-6 in collected supernatants were tested by ELISA kits.

#### Western Blot Analysis

The ANA-1 cells were plated in a 6-well plate as 1×10^6^ cells/well for 24 h. Cells were treated with XPS, XPS5-1 (both 100 μg/ml), and DEX (20 μM) for another 24 h after LPS (0.5 μg/ml) stimulation. Cells were collected to detect the expression of inflammation-related proteins (*n* = 4 for each group).

Total cell lysates were centrifuged at 4°C (2000 g, 15 min). Samples were loaded on 12% SDS-PAGE gel and were transferred to PVDF membranes. After blocking, the membranes were incubated with antibodies against iNOS, arginase-1, and GAPDH (all at 1:1,000 dilution) for 10 h at 4°C and secondary antibody HRP-conjugated IgG (1:2000) for 2 h at room temperature. The protein bands were visualized by the enhanced chemiluminescence (ECL) method and quantified by the ImageJ software. The GAPDH protein was served as internal control (Feng et al., 2019).

#### Immunofluorescence Confocal Microscopy

ANA-1 cells were cultured on a 24-well plate and then administrated with XPS, XPS5-1 (both 100 μg/ml), and DEX (20 μM) for another 24 h after LPS (0.5 μg/ml) was stimulated. Each representative experiment has three independent biological replicates.

After being fixed in 4% paraformaldehyde and permeated with 0.3% Triton/PBS, the cells were incubated at 4°C with iNOS (1:500) and arginase-1 (1:200) antibodies for 12 h. Then they were incubated with fluorescent dyes conjugated secondary antibodies (green for arginase-1 and red for iNOS) for 1 h at room temperature. Nuclei were observed after staining with DAPI at 10 μg/ml for 15 min. The cells were immediately observed by a fluorescence microscope (Leica EL6000 Microsystems) (He et al., 2020).

#### Flow Cytometry Analysis of Macrophages Phenotype *In Vitro*

ANA-1 cells were cultured as previously described and expression of CD11b^+^, F4/80^+^, CD86^+^, and CD206^+^ was detected (Wang B. et al., 2020). ANA-1 cells were all marked with fluorescently labeled antibodies targeting F4/80 and CD11b. To characterize the M1 phenotype, cells were marked with a CD86 antibody. To characterize the M2 phenotype, cells were marked with a CD206 antibody (Geng et al., 2020). Then the data (*n* = 4 for each group) were analyzed using the FlowJo software (Ashland, OR, United States).

### Evaluation of *In Vivo* Anti-Inflammatory Activity of XPS

#### Animals and Ethics Statement

Six- to eight-week-old male BALB/C mice were obtained from the Shanghai SLAC Lab Animal Co. Ltd. All animal protocols in this experiment met the dictates of the National Animal Welfare Law of China and were in agreement with the Animal Ethical Committee of School of Pharmacy, Fudan University (approved identification: 2020-01-YL-LH-01).

#### Experimental Design of LPS-Induced ALI Model

Mice were randomly divided into six groups (*n* = 6 for each group): sham, LPS-stimulated (LPS 3 mg/kg, model), LPS + XPS (20, 40, and 80 mg/kg), and LPS + DEX (4 mg/kg) groups. The mice were intratracheally instilled with normal saline (NS, the sham group) or LPS. ALI mice were treated 2 h after instillation. The mice in the sham and model group were given NS, and the mice in the LPS + XPS groups were administered with XPS intragastrically. DEX was administered intravenously as a positive control. All mice were sacrificed by an overdose of urethane 24 h after treatment (Xu et al., 2015).

#### Histopathologic Evaluation

The upper right lung lobe was chosen for histological examination. The tissues were fixed in 4% formaldehyde and embedded in paraffin. The embedded sections (5 μm thick) were stained with hematoxylin and eosin (H&E) solution to perform histopathological assessments (Wang et al., 2020d). The pathological ALI scoring system from the American Thoracic Association was used to score lung injury in a blinded manner (Matute-Bello et al., 2011). The scoring was assessed from 0 (normal) to 2 (severe) for the following categories: PMNs or monocytes in the interstitial space and alveolar septal thickening. The injury score was calculated according to the formula [(neutrophils in the interstitial space × 14) + (alveolar septal thickening × 2)]/(number of fields × 100) (Matute-Bello et al., 2011).

#### Bronchoalveolar Lavage Fluid Acquisition and Measurement of the Numbers of Mononuclear Cells and the Protein Level

After mice were sacrificed, trachea cannula was used to get the bronchoalveolar lavage fluid (BALF) from the left lungs. Collected lavage was centrifuged at 80 g for 15 min and retained for subsequent tests at −80°C.

The protein concentration in BALF was tested by the BCA assay kit. The mononuclear cells were counted with a hemocytometer (Jia et al., 2014).

#### MPO and Inflammatory Cytokines Assay in Lung

MPO is a mark of neutrophils infiltration in the lungs (Ding et al., 2019). The MPO concentration in the BALF was assayed by the ELISA kit. The levels of cytokines in BALF such as IL-1β, TNF-α, IL-6, and IL-10 were detected by ELISA kits.

#### Immunohistochemistry

The detection of immunohistochemical staining was performed according to our previous research (Kang, 2018). The slices were incubated with F4/80 antibody (1:100) to detect lung macrophages or Ly-6G/Ly-6C (Gr-1) antibody (1:100) to label polymorphonuclear leukocytes for 12 h at 4°C. The next day, HRP-labeled secondary antibodies (1:2000) were added and incubated at 37°C for 1 h. The sections were stained with the DAB solution and hematoxylin. The average optical density (AOD) of the positive area in lung tissue was measured as previously described (Zheng et al., 2020). AOD = IOD (Integrated Optical Density)/Area.

#### Western Blot Analysis

Lung tissues were homogenized and extracted with RIPA lysis reagent. The samples were loaded on gel for electrophoresis and transformed to PVDF membranes. After blocking, the membranes were incubated with primary antibodies against TLR4, TRAF6, MyD88, NF-κB p65, NF-κB p-p65, iNOS, arginase-1, and GAPDH (all at 1:1,000 dilution) for 10 h at 4°C and anti-rabbit secondary antibody HRP-conjugated IgG (1:2,000) at room temperature for 2 h.

### Statistics

Values were expressed as means ± SD. Multiple group comparisons were performed using one-way analysis of variance (ANOVA) followed by Bonferroni *post hoc* test using the software of SPSS. *p*-value of less than 0.05 (*p* < 0.05) was considered to be significant.

## Results

### Extraction, Isolation, and Purification of Glycoproteins XPS and XPS5-1

**FIGURE 1:**
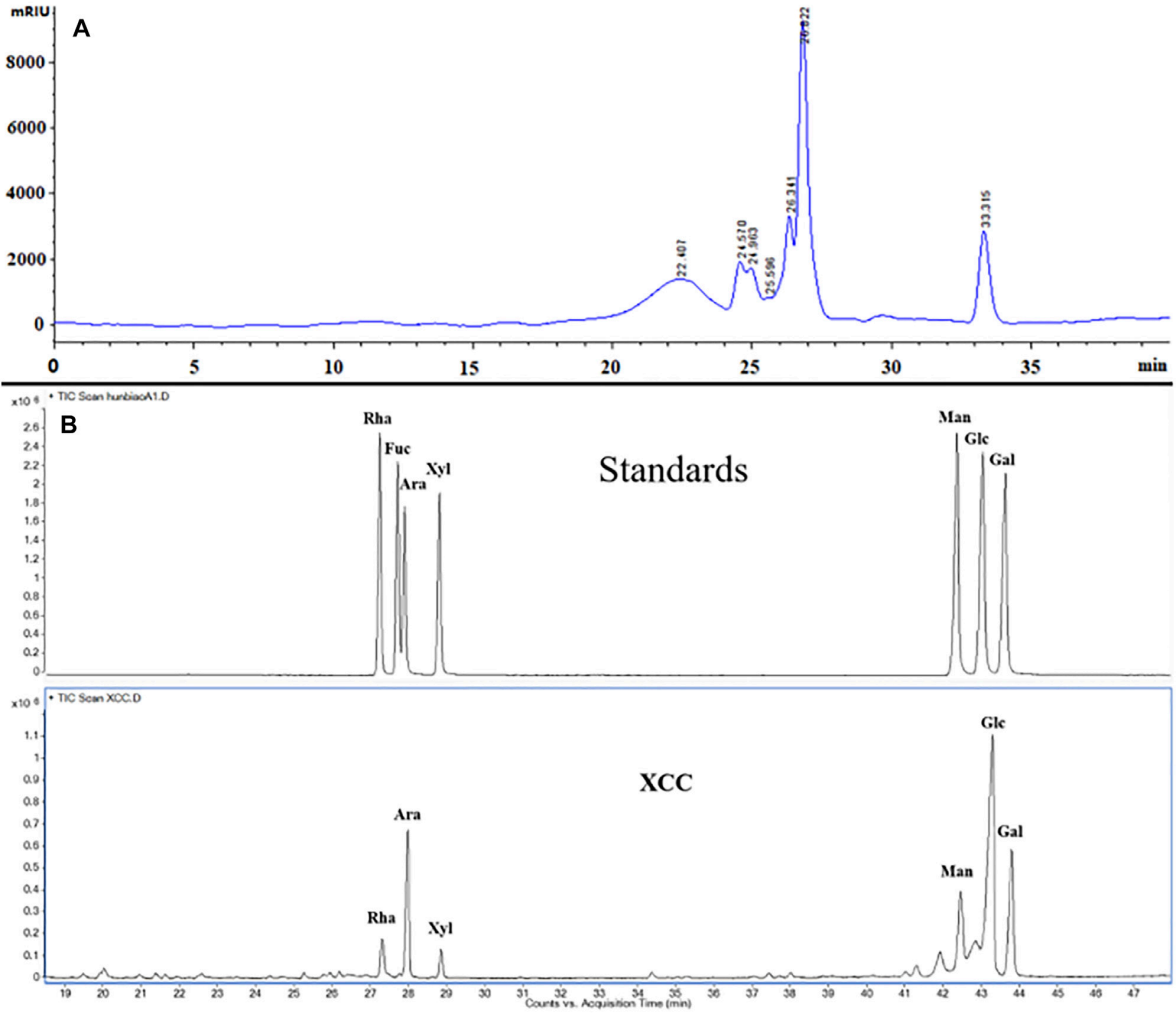
Characterization of XPS by HPGPC chromatogram and monosaccharide composition. **(A)** HPGPC chromatogram of XPS. **(B)** Monosaccharide composition analysis of XPS by GC.

315 g glycoproteins XPS (yield 6.3% of 5.0 kg dried material) were obtained from the overground part of *R. japonica* var. *glaucocalyx* by boiling water extraction and were characterized (Figure 1). XPS and XPS5 were isolated by a DEAE-cellulose column. XPS5 was further purified on a column of Superdex-75 to give XPS5-1. The total carbohydrate content and protein content of XPS were 20.61 ± 0.38% and 60.08 ± 1.13%, respectively. XPS contained 10.96 ± 1.68% of uronic acid determined by the m-hydroxyl biphenyl method. Monosaccharide composition analysis showed that XPS had glucose, galactose, arabinose, mannose, rhamnose, and xylose with the molar ratio of 10.0, 3.8, 3.1, 1.2, and 0.6. XPS5-1, with Mw estimated to be 3.17 × 10^3^ Da, and contained rhamnose, glucose, and arabinose with the molar ratio of 10.0:3.5:0.9, and amino acid of Glu:Ser:Gly = 30.2:21.6:48.2; the content of protein in XPS5-1 was 49.9% (Wang et al., 2020a). The profile of XPS5-1 had been published in another article (Wang et al., 2020a).

### Anti-Inflammatory Activity *In Vitro*

#### Effects of XPS and XPS5-1 on Cell Viability and Production of Inflammatory Mediators *In Vitro*

**FIGURE 2:**
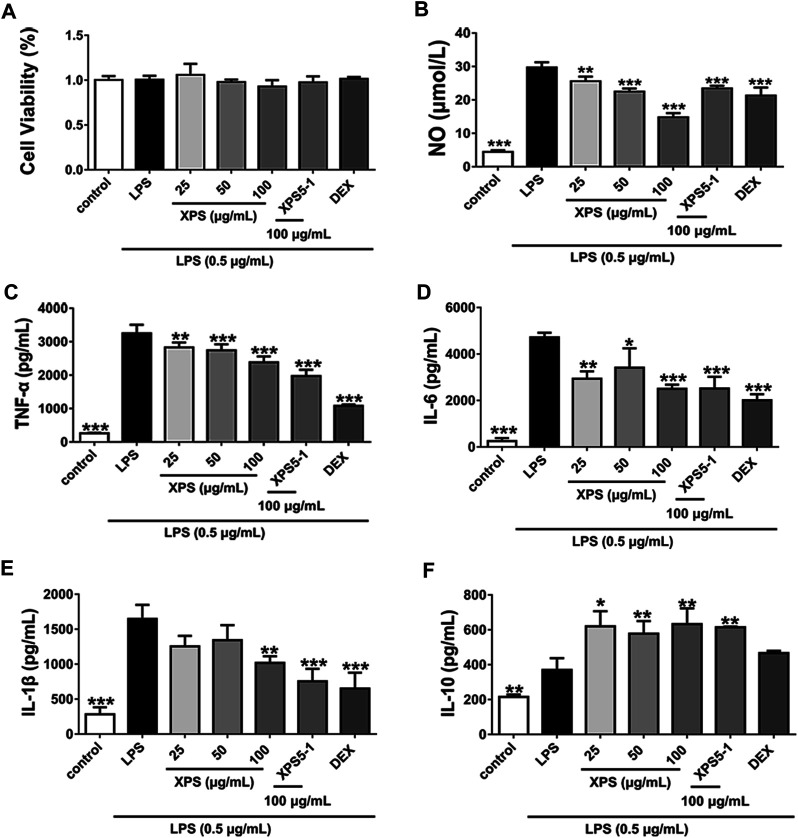
Effects of XPS and XPS5-1 on LPS-induced inflammatory response in ANA-1 cells. Cells were stimulated by LPS (0.5 μg/ml) with the treatment of XPS (25, 50, and 100 μg/ml), XPS5-1 (100 μg/ml), and DEX (20 μM) for 24 h. The cell viability was measured **(A)**. The levels of NO **(B)**, TNF-α **(C)**, IL-6 **(D)**, IL-1β **(E)**, and IL-10 **(F)** in the supernatant were detected (*n* = 3, means ± SD). ^*^ *p* < 0.05, ^**^ *p* < 0.01, and ^***^ *p* < 0.001 vs. the LPS-stimulated group, analyzed by ANOVA and Bonferroni *post hoc* test.

XPS, XPS5-1, and DEX had no effect on cell viability after LPS stimulation (Figure 2A). Furthermore, stimulation with LPS observably increased the levels of NO, TNF-α, IL-6, and IL-1β (*p* < 0.001, Figures 2B–E). The level of inflammatory mediators, including NO, TNF-α, and IL-6, was significantly decreased by XPS treatment (*p* < 0.05). The level of IL-1β was reduced by 100 μg/ml XPS treatment (*p* < 0.01). The production of IL-10 distinctly rose by LPS stimulation (*p* < 0.01, Figure 2F), and XPS further elevated it (*p* < 0.05). DEX markedly decreased the level of NO, TNF-α, IL-6, and IL-1β (*p* < 0.001) but had less effect on IL-10 production. The effect of XPS5-1 (100 μg/ml) was similar to XPS.

#### Effects of XPS and XPS5-1 on Macrophage Polarization

**FIGURE 3:**
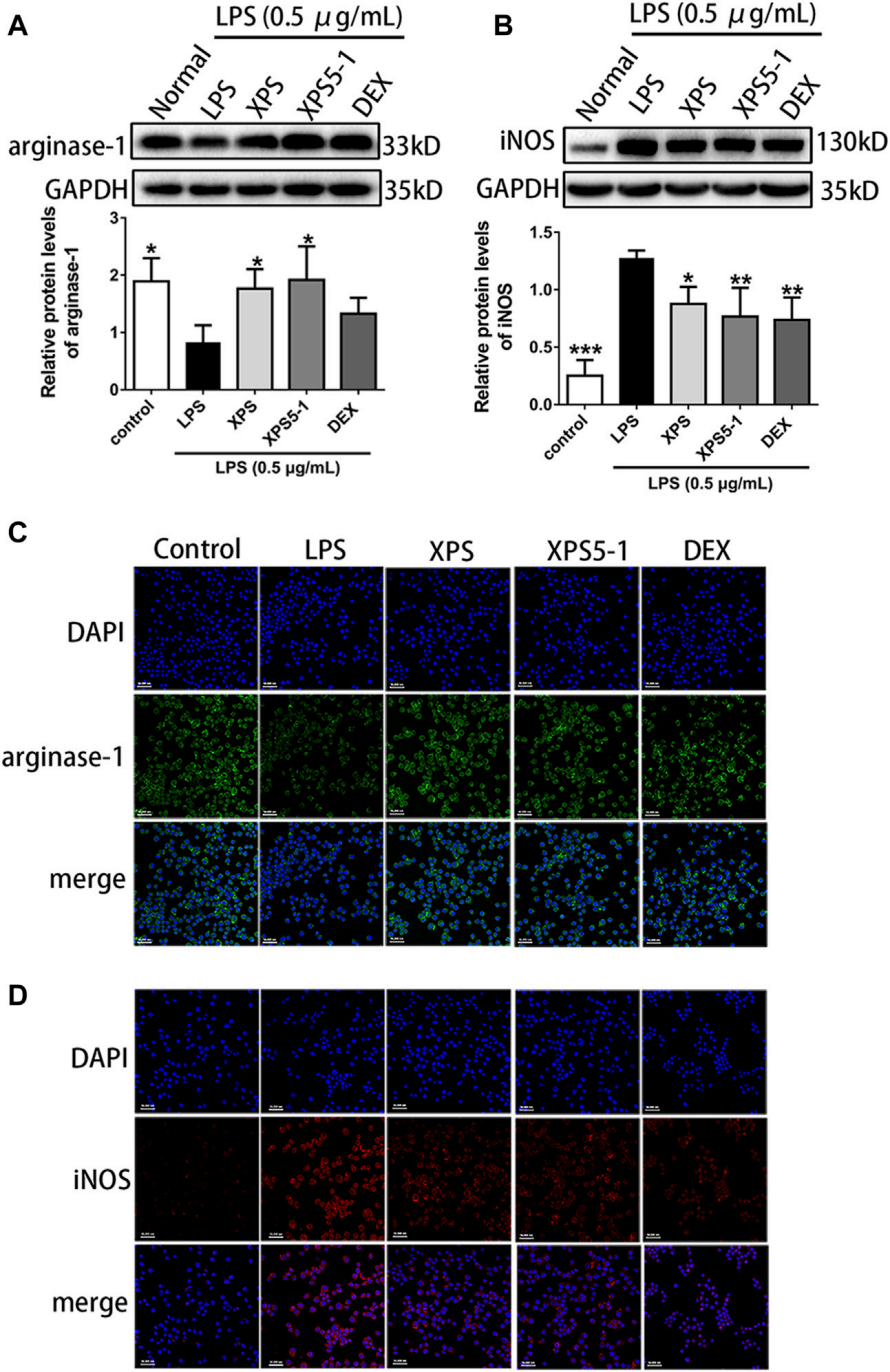
Effects of XPS and XPS5-1 on protein expression in LPS-stimulated ANA-1 cells. Total cell lysates were collected for analyzing the expression of arginase-1 **(A)** and iNOS **(B)** by Western blot (*n* = 4, means ± SD). The effects of XPS, XPS5-1 (both 100 μg/ml), and DEX (20 μM) on the expression of arginase-1 (green) and iNOS (red) were detected by immunofluorescence, and cell nuclei were in blue **(C, D)**. ^*^ *p* < 0.05, ^**^ *p* < 0.01, and ^***^ *p* < 0.001 vs. the LPS-stimulated group, analyzed by ANOVA and Bonferroni *post hoc* test.

The location and expression level of proteins were tested by immunofluorescence assay and Western blot. Arginase-1 was normally expressed in the control group, whereas LPS obviously reduced its expression. As an inducible synthase, iNOS was less expressed in resting cells. The expression of it increased after LPS stimulation (*p* < 0.001, Figure 3).

After administration, XPS and XPS5-1 markedly upregulated the level of arginase-1 (*p* < 0.05, Figure 3A), but DEX only had a mild effect on arginase-1 expression. The intracellular green fluorescence (represented arginase-1) was bright in the XPS and XPS5-1 groups.

XPS, XPS5-1, and DEX distinctly downregulated the expression of iNOS (*p* < 0.05, Figure 3B). The intracellular red fluorescence (labeled iNOS) was dim in the XPS, XPS5-1, and DEX groups.

**FIGURE 4:**
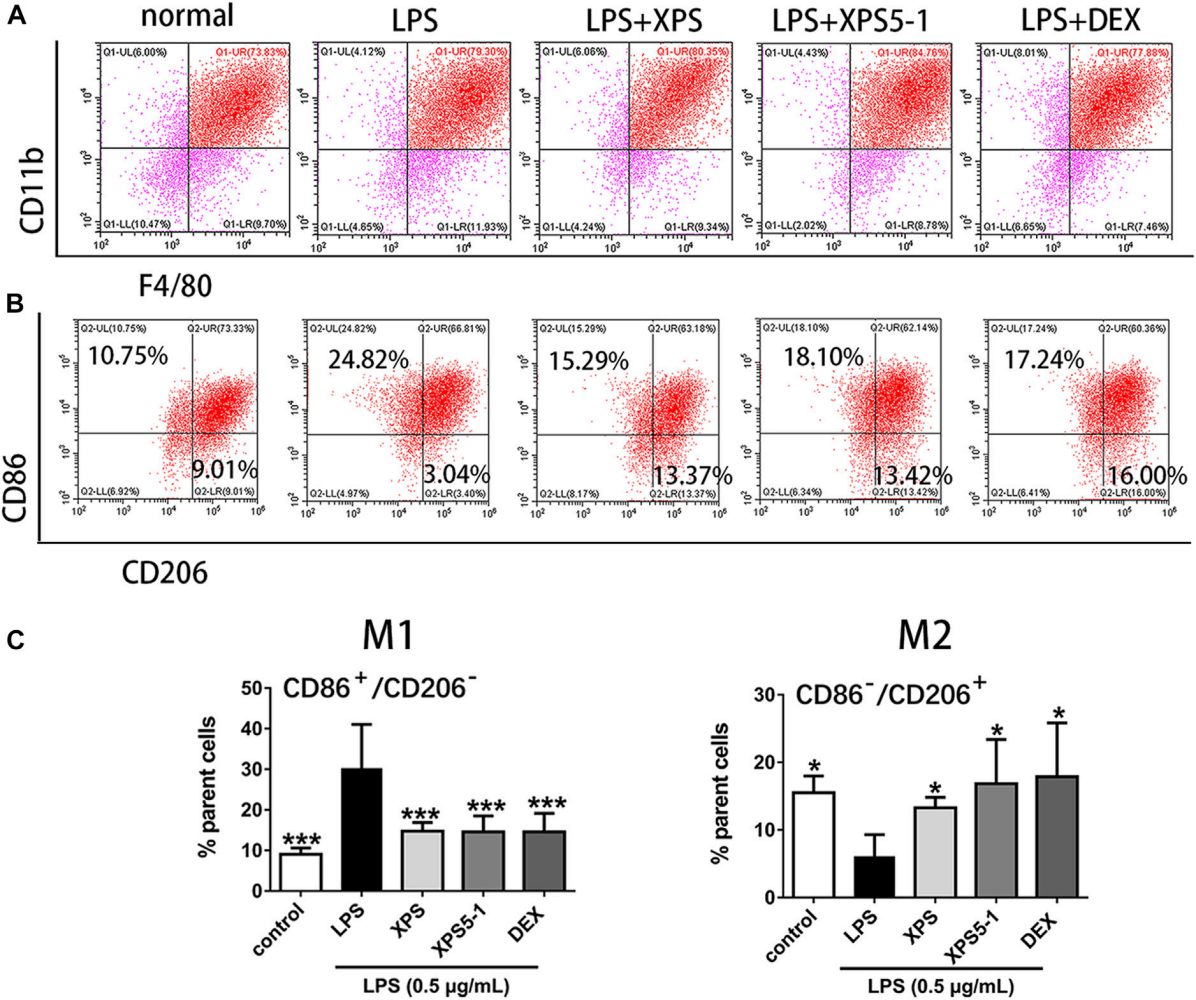
Effect of XPS and XPS5-1 on LPS-induced macrophage polarization in ANA-1 cells. **(A)** Dot plots showing macrophages. **(B)** CD86^+^/CD206^–^ (upper left area representing M1) and CD86^–^/CD206^+^ (lower right area representing M2) by flow cytometry. **(C)** Graphs showing the radio of these two phenotypes. ANA-1 cells were stimulated by LPS (0.5 μg/ml) with XPS, XPS5-1 (both 100 μg/ml), and DEX (20 μM) for 24 h. The cells were incubated with fluorescently labeled antibodies. The expression ratio was detected by flow cytometry (*n* = 4, means ± SD). ^*^ *p* < 0.05, ^**^ *p* < 0.01, and ^***^ *p* < 0.001 vs. the LPS-stimulated group, analyzed by ANOVA and Bonferroni *post hoc* test.

After LPS stimulation, the macrophage phenotype of ANA-1 cells was detected by flow cytometry. Double-positive of F4/80 and CD11b indicated that they belong to the macrophage lineage (Figure 4A).

After 24 h of culture, LPS stimulation promoted a proinflammatory (M1) phenotype of macrophages, characterized by the increased events of CD86^+^/CD206^–^ (M1) in ANA-1 (*p* < 0.001). XPS and XPS5-1 obviously reversed the LPS-promoted M1 subtype (*p* < 0.001). The cells stimulated by LPS expressed less CD86^–^/CD206^+^ events (M2) (*p* < 0.001). XPS and XPS5-1 administration skewed the LPS simulated ANA-1 to an anti-inflammatory (M2) phenotype, characterized by the increased events of CD86^–^/CD206^+^ (*p* < 0.05, Figures 4B,C).

### Anti-Inflammatory Effects of XPS *In Vivo*

#### Effects of XPS on LPS-Induced ALI

**FIGURE 5:**
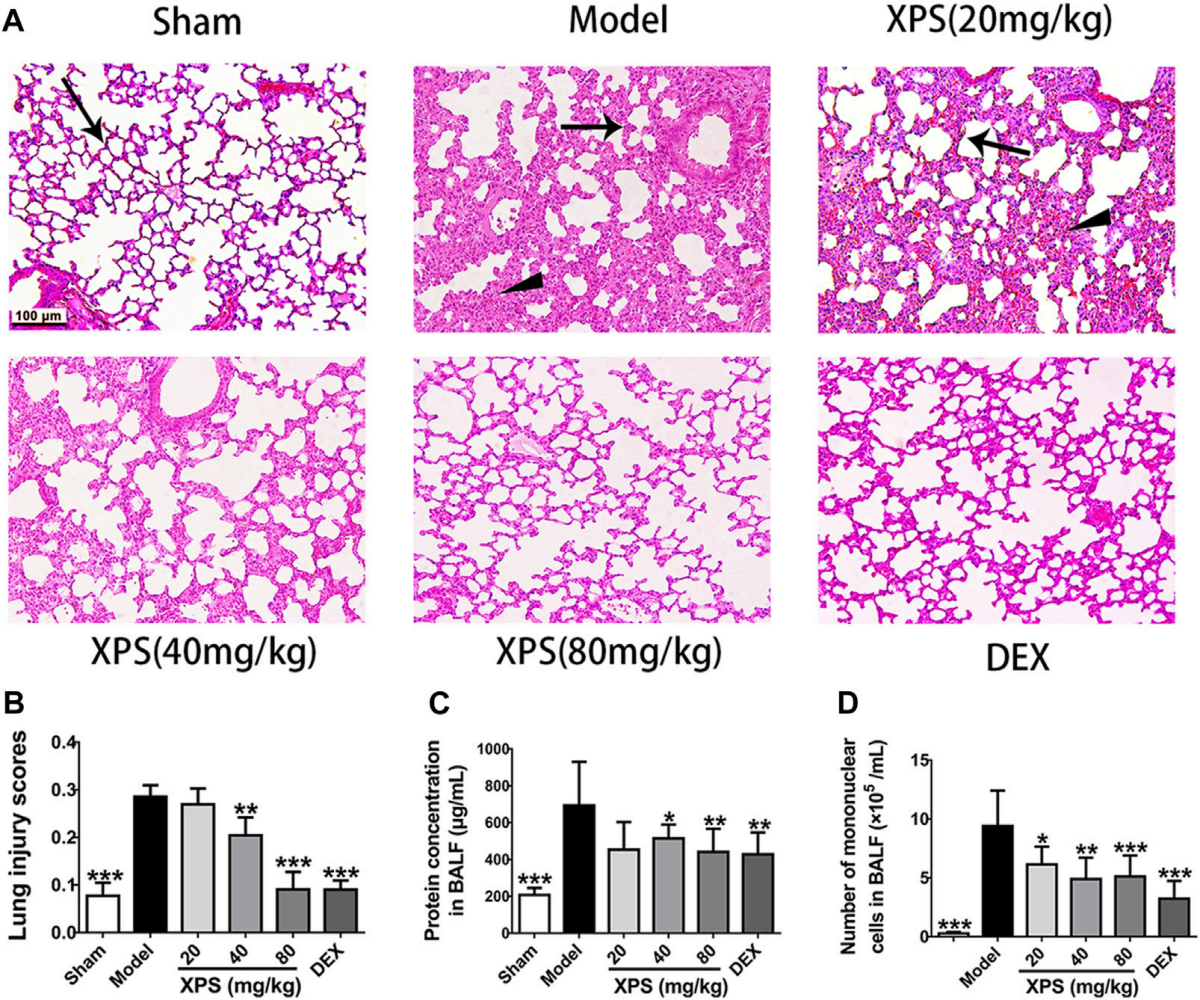
XPS alleviated lung injury in ALI mice. H&E-stained lung tissues were observed under light microscopy 200× magnification. Wedge indicates accumulation of mononuclear cells, and black arrow indicates the alveolar wall **(A)**. Lung injury scores were assessed **(B)**. The protein concentration **(C)** and the numbers of mononuclear cells **(D)** in BALF were detected 24 h after XPS administration (*n* = 6, means ± SD). ^*^ *p* < 0.05, ^**^ *p* < 0.01, and ^***^ *p* < 0.001 vs. the model group, analyzed by ANOVA and Bonferroni *post hoc* test.

To estimate the effect of XPS treatment in LPS-stimulated mice at histological level, the lung tissues were tested by H&E staining. The sham group showed only mild changes caused by intratracheal instillation of NS. However, pulmonary congestion, alveolar wall thickness, and edema were observed in the model group. XPS or DEX administration obviously alleviated these damages (Figure 5A). The LPS group had the highest lesion scores and the XPS or DEX group had lower scores, which reflected the levels of lung injury (Figure 5B).

To further assess the extent of alveolus damage, the BALF was collected to test the protein concentration and the mononuclear cell counts. In comparison with the sham group, dramatic increases were observed in protein concentration and cell counts after LPS instillation (*p* < 0.001). XPS (40 and 80 mg/kg) and DEX (4 mg/kg) treatment significantly restrained these phenomena (*p* < 0.05, Figures 5C, D).

#### XPS Attenuated Accumulation of Inflammatory Cells in the Lung

**FIGURE 6:**
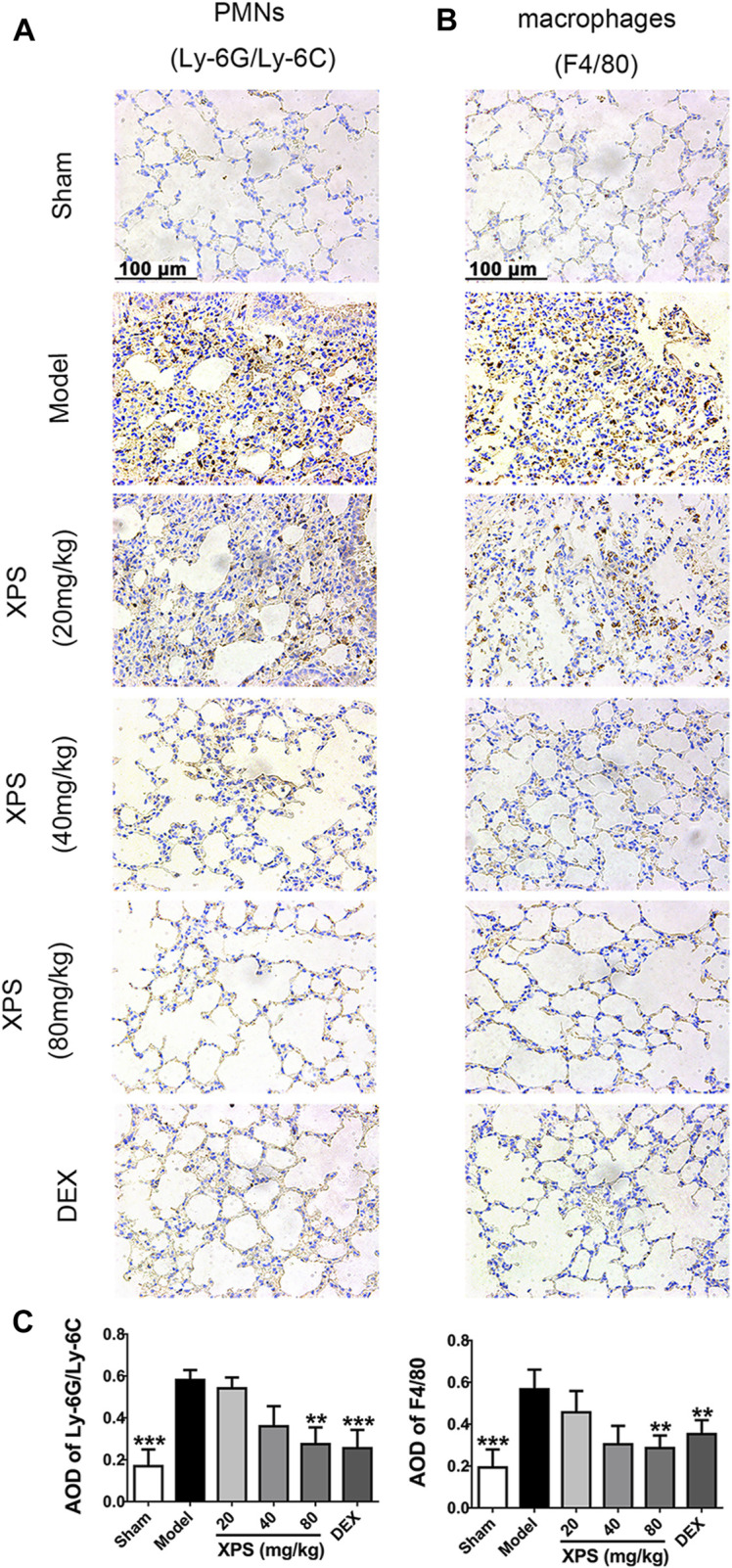
XPS inhibited inflammatory cells infiltration in lung tissue of ALI mice. The PMNs **(A)** and macrophages’ **(B)** infiltration were observed under light microscopy (400×) by immunohistochemistry analysis. A semiquantitative analysis of the images **(C)** was performed by measuring the AOD (*n* = 4, means ± SD). ^*^ *p* < 0.05, ^**^ *p* < 0.01, and ^***^ *p* < 0.001 vs. the model group, analyzed by ANOVA and Bonferroni *post hoc* test.

The effect of XPS on attenuating the accumulation of macrophages (expressing F4/80) and PMNs (expressing Ly-6G/Ly-6C (Gr-1)) in lung tissue was evaluated (Figures 6A,B). The results showed a high level of PMNs and macrophages in the model group. The XPS and DEX groups exhibited lower levels of staining in lung tissue compared with the model group. These observations were also supported by the semiquantitative analysis. XPS (40 and 80 mg/kg) and DEX (4 mg/kg) obviously reduced the infiltration of inflammatory cells (*p* < 0.01, Figure 6C).

#### XPS Regulated the Levels of Inflammatory Cytokines and MPO in BALF

**FIGURE 7:**
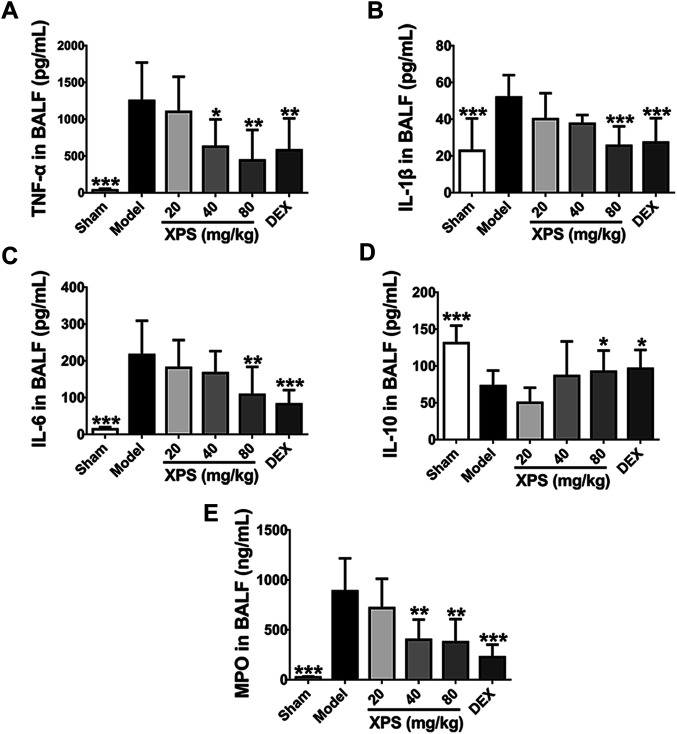
XPS regulated the level of inflammatory cytokines and MPO in BALF. The levels of TNF-α **(A)**, IL-1β **(B)**, IL-6 **(C)**, IL-10 **(D)**, and MPO **(E)** in BALF were tested by ELISA (*n* = 6, means ± SD). ^*^ *p* < 0.05, ^**^ *p* < 0.01, and ^***^ *p* < 0.001 vs. the model group, analyzed by ANOVA and Bonferroni *post hoc* test.

Compared with the sham group, the level of TNF-α, IL-1β, and IL-6 was obviously elevated in the model group (*p* < 0.001, Figures 7A–C). XPS (80 mg/kg) and DEX significantly attenuated the levels of these proinflammatory cytokines (*p* < 0.01). XPS (40 mg/kg) only markedly inhibited the expression of TNF-α (*p* < 0.05). XPS 20 mg/kg mildly inhibited the release of cytokines with no significance. The production of IL-10 distinctly decreased in the model group (*p* < 0.001, Figure 7D). XPS (80 mg/kg) and DEX significantly increased the level of IL-10 (*p* < 0.05). Treatment of XPS in 20 and 40 mg/kg only showed an upward tendency with no significance.

MPO is a reliable marker for neutrophils infiltration and catalyzes oxidants generation (Lei et al., 2020). Compared with the sham group, MPO level was obviously increased in the model group (*p* < 0.001), while XPS (40 and 80 mg/kg) and DEX markedly reduced such elevations (*p* < 0.01, Figure 7E).

#### XPS Inhibited the Expression of Proteins in TLR4/MyD88 Signaling Pathway

**FIGURE 8:**
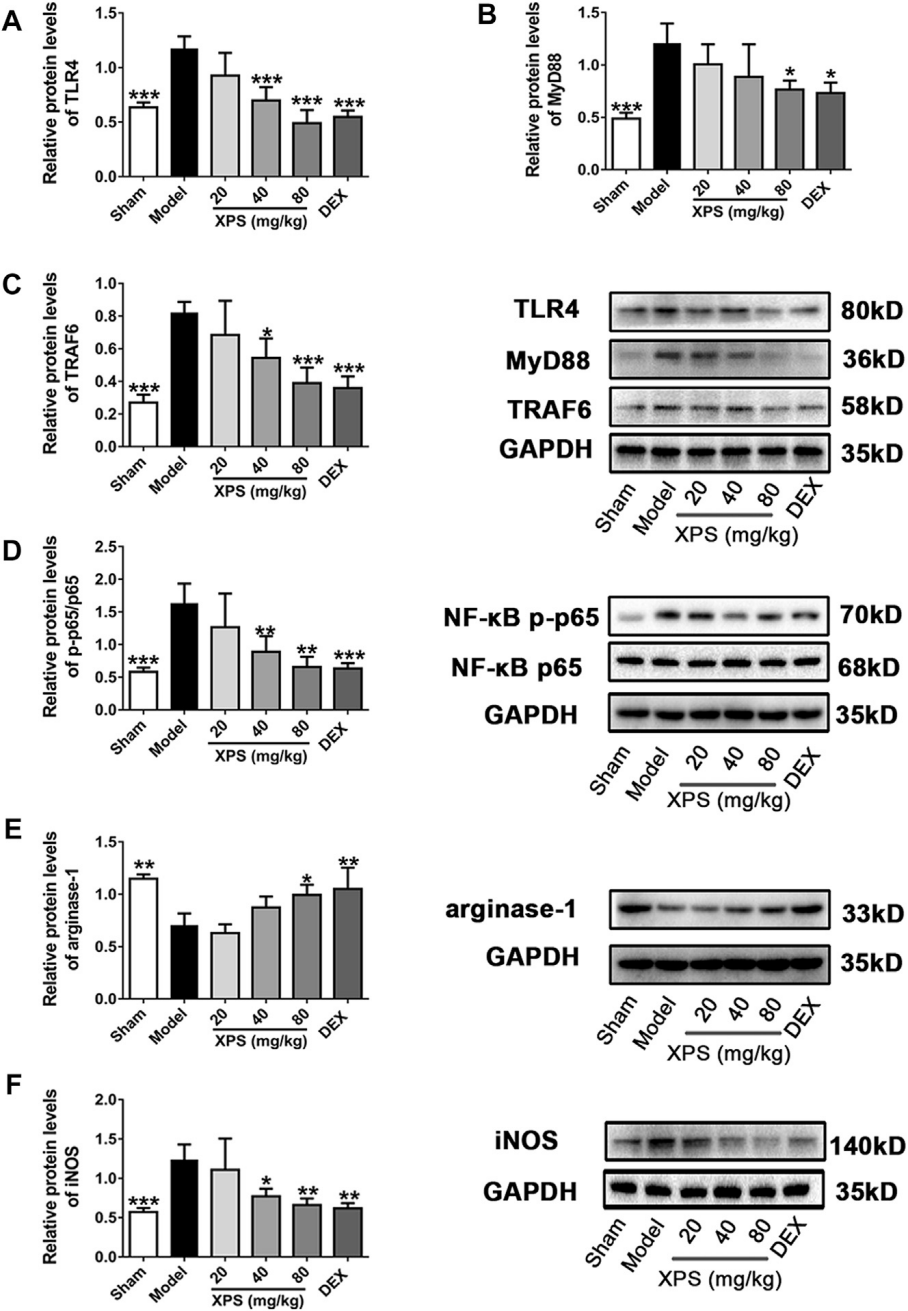
XPS downregulated the TLR4/MyD88 signaling pathway in the lung. Lung tissues were harvested for Western blot analysis (*n* = 4, means ± SD). The protein expression of TLR4 **(A)**, MyD88 **(B)**, and TRAF6 **(C)** and the ratio of NF-κB p-p65 to NF-κB p65 **(D)** were shown. The level of arginase-1 **(E)** and iNOS **(F)** was detected. ^*^ *p* < 0.05, ^**^ *p* < 0.01, and ^***^ *p* < 0.001 vs. the model group, analyzed by ANOVA and Bonferroni *post hoc* test.

LPS obviously augmented the expression of TLR4, MyD88, and TRAF6 in the model group compared with the sham group (*p* < 0.001, Figures 8A–C). XPS (40 and 80 mg/kg) decreased the protein content of TLR4 and TRAF6 (*p* < 0.05), while only XPS 80 mg/kg significantly reduced MyD88 expression (*p* < 0.05).

The phosphorylation level of NF-κB p65 in the cytosol was expressed by the ratio of NF-κB p-p65 to NF-κB p65. The ratio (Figure 8D) was markedly elevated in the model group (*p* < 0.001) and was downregulated by XPS (40 and 80 mg/kg, *p* < 0.01). Arginase-1 expression reduced markedly in the model group (*p* < 0.01, Figure 8E) but was elevated by XPS (80 mg/kg, *p* < 0.05). Moreover, iNOS (Figure 8F) expression was consistent with the phosphorylation level of NF-κB. Similarly, XPS (40 and 80 mg/kg) decreased the expression of iNOS (*p* < 0.05).

## Discussion

*R. japonica* var. *glaucocalyx* is a TCM prescribed to treat inflammatory diseases. The terpenoid and flavonoid in *R. japonica* var. *glaucocalyx* show the anti-inflammatory activity (Wang et al., 2020a), but the effect of macromolecules in this plant is unclear. In recent years, lots of researches have focused on the anti-inflammatory and antitumor effects of glycoproteins due to their remarkable therapeutic effects and relatively low toxicity (Fu et al., 2021).

XPS was glycoproteins isolated from *R. japonica* var. *glaucocalyx*. XPS5-1 and XPS10-1 were glycoproteins purified from XPS. XPS5-1 showed an inhibitory effect *in vitro* on tryptophan 2,3-dioxygenase (TDO) (Wang et al., 2020a), an enzyme that subsequently mediates the immune response to cancers. XPS10-1 is an inhibitor of indoleamine 2,3-dioxygenase (IDO) (Wang et al., 2020b). However, our preliminary experiment found that only XPS and XPS5-1 had anti-inflammatory activity. The present work demonstrated that XPS and XPS5-1 inhibited inflammatory response *in vitro* through modulating macrophage polarization with the reduction of inflammatory mediators.

Macrophages have been widely studied as the center of inflammatory response in recent years (Anders et al., 2018). Macrophages can be grouped into two main phenotypes, M1 or M2, depending on the various stimuli and specific microenvironment of tissue (Ye W. et al., 2020). The activated M1 phenotype is associated with the ability to release IL-1β, TNF-α, IL-6, and NO (Askari and Shafiee-Nick, 2019). Additionally, M2 macrophages upregulate the immune-suppressive factors and anti-inflammatory cytokines, including arginase-1 and IL-10 (Su et al.).

Arginase-1 and iNOS are involved in two opposite macrophage phenotypes, M1 and M2 (Orecchioni et al., 2019). Both enzymes utilize L-arginine (L-Arg) as a substrate and are mutually downregulated (Liu L. L. et al., 2019). The iNOS is typically elevated in inflammatory states and catalyzes the production of NO (Wang et al., 2017). The excessive production of NO not only is helpful for fighting pathogens but also leads to inflammation and organs injury (Cinelli et al., 2020).

LPS as bacteria endotoxin polarized macrophage to M1 with more expression of CD86^+^ and less CD206^+^ (Anders et al., 2018). It also increased iNOS expression but decreased the level of arginase-1. XPS and XPS5-1 treatment significantly reversed these phenomena. The LPS-induced proinflammatory cytokine (TNF-α, IL-1β, and IL-6) production and anti-inflammatory cytokine (IL-10) level were adjusted by XPS and XPS5-1 treatment. All the results suggested that the anti-inflammatory effects of XPS and XPS5-1 might be mediated by promoting macrophage polarization from M1 to M2 *in vitro*.

According to our results, XPS accounted for 6.3% of the plants, and XPS5-1 yield rate from XPS was 0.82%. XPS was easier to obtain considering the isolation and purification process. XPS5-1 has similar anti-inflammatory effects as XPS *in vitro*; it may be established as the quality control indicator of XPS. The anti-inflammation effect of XPS was further detected in LPS-induced ALI mice.

Immune cells in the lung such as alveolar macrophages and PMNs further cause a proinflammatory microenvironment and aggravate lung injury (Mantovani et al., 2019). The superfluous recruitment of PMNs into lung tissues is a hallmark of ALI (Peng et al., 2019; Ye S. et al., 2020). MPO is an enzyme present only in PMNs and involves oxidative stress (Ye S. et al., 2020). A high level of MPO in the lung represents the infiltration of PMNs, which could accelerate the development of ALI (Ding et al., 2019).

XPS administration obviously decreased the number of mononuclear cells in BALF and inhibited PMN infiltration with the reduction of MPO level. The results in the immunohistochemical analysis also demonstrated that XPS treatment inhibited neutrophils and monocytes infiltration.

Enhanced macrophage M1 polarization relates to the inflammation in the lungs. When M1 phenotype macrophages are constantly activated, the proinflammatory cytokines, such as TNF-α, IL-1β, and IL-6, are released. Activated M2 phenotype macrophages produce IL-10, a potentially immunosuppressive cytokine, to terminate inflammatory responses and participate in tissue repair (Su et al.).

After LPS stimulation, TNF-α is produced first and upregulates the secretion of IL-1β (Zhao et al., 2019). As an amplifier in the inflammatory response, IL-1β increases pulmonary vascular permeability and is widely accepted as an important molecular marker for the ALI (Mantovani et al., 2019). The high level of IL-1β and TNF-α increases the expression of IL-6, a pleiotropic cytokine involved in chronic inflammation, which may promote the migration of neutrophils and contribute to organ damage (Fields et al., 2019). IL-10 production decreased in the ALI model group, which also represents a hyperinflammatory state (Su et al.).

XPS administration obviously decreased the production of TNF-α, IL-1β, and IL-6 with the increased production of IL-10 in the lung of ALI mice. XPS treatment alleviated the damages of the lung caused by excessive inflammation.

LPS-TLR4/NF-κB signaling pathway is essential in inflammatory response (Liu J. et al., 2019). The myD88-dependent pathway is important in pulmonary inflammation and is required for the infiltration of neutrophils (Wang et al., 2020b). LPS activates TRAF6 via TLR4 and MyD88 and then phosphorylates NF-κB. Subsequently, large amounts of inflammatory cytokines mentioned above are released (Zhou et al., 2019).

The expression of iNOS is motivated by the activation of the TLR4/NF-κB pathway (Zhou et al., 2019). A large amount of NO produced by iNOS in the lung accelerates the recruitment and infiltration of mononuclear cells in the lung of ALI (Hu et al., 2020).

Our testing results indicated that LPS stimulation upregulated the expression of TLR4, MyD88, TRAF6, and p-NF-κB. XPS administration decreased the expression of key proteins. The excessive iNOS and proinflammatory cytokines were reduced by XPS treatment. XPS might alleviate ALI by downregulating the LPS-TLR4/NF-κB signaling pathway.

*R. japonica* var. *glaucocalyx* has been traditionally used in numerous inflammatory diseases. Our research suggested that the activity of *R. japonica* var. *glaucocalyx* might partly come from glycoproteins (XPS), an important ingredient in herbs. As homogeneous glycoproteins purified from XPS, XPS5-1 had similar activity *in vitro*. Consistent with the traditional application of *R. japonica* var. *glaucocalyx*, XPS might be a promising candidate for inflammatory diseases, especially for treating ALI.

## Conclusion

*In vitro,* XPS and its purified ingredients (XPS5-1) inhibited the secretion of proinflammatory mediators and polarized LPS-stimulated macrophages from M1 to M2. *In vivo,* XPS ameliorated LPS-induced ALI by reducing the inflammatory response and inhibiting macrophages and PMNs accumulation in lung tissues. These phenomena were related to the inhibition of the TLR4/NF-κB pathway. Glycoproteins from *R. japonica* var. *glaucocalyx* could be a potential agent for ALI.

## Data Availability

The original contributions presented in the study are included in the article/supplementary material; further inquiries can be directed to the corresponding authors.
